# Impact of Immediate Dentin Sealing Using Universal Adhesive under Simulated Pulp Pressure on Microtensile Bond Strength of Indirect Resin Composite Restorations and Dentin Permeability

**DOI:** 10.1055/s-0041-1735442

**Published:** 2021-11-16

**Authors:** Emad Abd Elfatah Abo-Alazm, Rehab Khalil Safy

**Affiliations:** 1 Department of Restorative Dentistry, Egyptian Russian University, Badr City, Cairo Governorate, Egypt; 2 Department of Restorative Dentistry, Faculty of Dentistry, Suez Canal University, El Salam District, Ismailia Governorate, Egypt

**Keywords:** CAD/CAM resin composite restorations, delayed dentin sealing, dentin permeability, immediate dentin sealing, microtensile bond strength, universal adhesive

## Abstract

**Objective** The aim of this study was to investigate the effect of immediate dentin sealing (IDS) technique using universal adhesive under simulated pulp pressure on microtensile bond strength (μTBS) of indirect resin composite restorations and dentin permeability.

**Materials and Methods** Fifty extracted caries-free human third molars were used for specimens' preparation. Each molar's occlusal table was abraded flat and their roots were separated under continuous water cooling. Forty specimens were used for microtensile bond strength test (μTBST) evaluation. The μTBST specimens were randomly assigned to two groups according to the dentin sealing time; Immediate dentin sealing (IDS) and delayed dentin sealing (DDS). Each group was further subdivided into two subgroups according to the adhesive system used for dentin sealing: iBOND self-etch adhesive and GLUMA Bond Universal. All specimens were exposed to simulated pulp pressure for 1 week then restored using computer-aided design/computer-aided manufacturing (CAD/CAM) resin composite blocks. The μTBS was evaluated for all tested subgroups after 24 hours and 6 months of water storage. The remaining 10 teeth were used for the preparation of dentin discs for dentin permeability evaluation. They were divided into two groups according to type of self-etch adhesive used. Fluid filtration rate was evaluated after etching, with smear layer and after adhesive application. Results obtained were statistically analyzed using Shapiro–Wilk test and Weibull analysis.

**Results** Statistically significant difference was recorded between µTBS mean values of both IDS and DDS techniques at 24 hours and after 6 months of water storage. GLUMA Bond Universal adhesive had significantly higher bond strength compared with iBond at both IDS and DDS techniques, but both adhesives showed a significant reduction in the Weibull characteristic strength after 6 months of water storage. Significant reduction in dentin permeability was recorded by both adhesives without any significant difference between them.

**Conclusions** The IDS technique using universal adhesive in self-etch mode is an effective strategy for improving the final bond strength of CAD/CAM resin composite restorations and reducing dentin permeability.

## Introduction

Indirect dental restorations have witnessed a massive increase in the use of computer-aided design and manufacturing (CAD/CAM) techniques over the last decade, likely due to impressive developments in intraoral imaging and manufacturing technologies. 1 Although the two major classes of CAD/CAM restorative materials are ceramics and composite resins, composite resin block materials offer major benefits attributed to their manufacturability, machinability, and repeatability. 1 2

Generally, adhesive–dentin interface is considered as the weak link of any bonded indirect restoration; therefore, microleakage and postoperative sensitivity remain a concern. 3 Desensitizing agents were suggested to be used in many adhesive restorative techniques as a routine practice to overcome the postoperative sensitivity. Nevertheless, some ingredients in dentine desensitizers may affect the sealing and bonding properties of luting agents. One of the most widely used desensitizers is a glutaraldehyde-based material (GLUMA desensitizer); it has been reported to reduce dentin permeability, and at the same time provides dentin disinfection; nevertheless, their effect on dentin bonding techniques remains debated. 4

Consequently, contemporary efforts have been performed to mitigate postoperative sensitivity through dentin sealing using available adhesive systems. 5 6 7 Dentin sealing procedure could be achieved, either, immediately after tooth preparation and before impression taking (IDS) or delayed (DDS). It has been proposed that IDS has the ability to decrease postoperative sensitivity and bacterial microleakage while improving bond strength of indirect restorations. So that, patients treated with IDS technique enjoyed better comfort during the provisional restoration stage, according to previous reports. 8 9 10 Self-etching adhesives were recently been used to improve IDS simplicity; it is one of the most auspicious due to less technique sensitivity through eliminating the etching and rinsing step. Furthermore, a crucial benefit of this approach is that the adhesive system is infiltrated simultaneously with the self-etching process, reducing the possibility of discrepancies between the two processes. 11 Recent adhesive technology research has also resulted in the development of multimode universal adhesives that could be used in either etch and rinse or self-etch depending on the application. Since their launch, the bonding effectiveness of universal adhesives has become a hot topic. 12 13 14

Although some *in vitro* studies have assessed the bond strength of universal adhesives, there is little data on how to achieve state-of-the-art dentin sealing of CAD/CAM resin composite restorations using self-etch mode of universal adhesive systems, which is becoming more common in clinical practice due to its reduced chair time and ease of use. It is also worth noting that, in vital teeth with positive pulpal pressure, dentinal fluid transudation through polymerized adhesive layers may prevent near-perfect dentin sealing while using simplified adhesive systems. In light of these considerations, the twofold purpose of this study was to evaluate the effect of IDS technique using self-etch mode of universal adhesive system on microtensile bond strength (μTBS) of indirect resin composite restorations and dentin permeability in presence of simulated pulpal pressure

Therefore, the null hypotheses tested in the current study were: (1) There is no difference in bond strength of IDS and DDS techniques either after 24 hours or 6 months of water storage, (2) There is no difference in bond strength exists when self-etch mode of universal adhesive and a self-etch adhesive used for IDS and DDS when tested after 24 hours and 6 months of water storage, and (3) there is no difference in the ability of both tested adhesive systems to reduce dentin permeability.

## Materials and Methods

This *in vitro* study was approved by the Research Ethics Committee (REC), Faculty of Dentistry, Suez Canal University (ethical approval No 250/2020). All tested materials and their description, composition, batch number, and manufacturer's information are displayed in Table 1 .

**Table 1:** Materials, description, composition, manufacturers, and batch numbers

Material	Description	Composition	Manufacturer	Batch Number
iBOND Self- Etch adhesive	A light-curing self-priming one component bonding agent	UDMA, 4-META, glutaraldehyde, acetone, water, photo initiators, stabilizers	Heraeus Kulzer, Hanau, Germany	010107
GLUMA Bond Universal	Light-curing self-priming one component bonding agent	UDMA, MDP phosphate monomers, 4-META, HEMA, acetone, water, photo initiators, stabilizers		010022 010022
ESPE SIL	Silane coupling agent	Ethyl alcohol, Methyl ethyl ketone methacryloxypropyl-trimethoxysilane	3M ESPE Germany	563857
RelyX Unicem shade A1	Dual-cure, self-adhesive universal resin cement	Powder: glass powder Initiator, silica, substituted pyrimidine, calcium hydroxide, peroxy compound pigment. Liquid: methacrylated phosphoric ester, dimethacrylate, acetate, stabilizer, initiator		426768
Cavex	Noneugenol temporary cement	Magnesium oxides, zinc oxides, fatty acid dimer, acetic acid	Cavex, RW Haarlem, Holland	50303
DiaTemp	Temporary filling material	Polyurethane dimethacrylate, hydrophilic methacrylate, nano silica and silver, catalysts, stabilizer	DiaDent, Buk-do, Korea	2001301
Grandio CAD/CAM restorative	Nanohybrid resin composite blocks, shade A3	Resin: Bis-GMA, TEGDMA. Filler: Ba–Al–Si glass/Silica nanoparticles 89% by weight and 71.4%by volume with a particle size range of 20–40 nm	VOCO GmbH, Germany	1702120

Abbreviations: 4-META, 4-methacryloyloxyethyltrimellitate anhydride; Bis-GMA, bisphenol A diglycidyl methacrylate; HEMA, 2-hydroxyethyl methacrylate; MDP, 10-methacryloyloxydecyl dihydrogen phosphate; TEGDMA, triethylene glycol dimethacrylate; UDMA, urethane dimethacrylate.

### Specimens Preparation

After sample size calculation, 50 human extracted crack and caries-free human mandibular third molars were extracted from patients aged between 18 and 25 years for the current study. All soft tissue remnants were removed, then teeth were stored in distilled water containing 0.2% thymol at room temperature for not more than 3 months at 4°C till testing. Each molar's occlusal table was abraded flat 1 mm behind the DEJ to reveal dentin surface with 600-grit SiC paper (3M of Brazil Ltd, Sumare, Brazil) under running water to create a standardized smear layer. Roots of each tooth were then separated by a diamond saw (IsoMet, 4000 Buehler, Lake Bluff, Illinois, United States) under continuous water cooling. Roots separation was achieved at a direction parallel to the occlusal surface 2 mm below the cementoenamel junction and pulp tissues were removed from the exposed pulp chambers. All specimens were checked for possible cracks at X20 magnification stereomicroscope (MA100 Nikon, Japan). Then, the flat dentin surfaces were then rinsed with water for 2 minute and blot dried. Specimens were randomly distributed through a research random assignment tool ( www.randomizer.org ): 40 for the microtensile bond strength test (µTBST) and 10 specimens for evaluation of dentin permeability.

### Microtensile Bond Strength Test

The µTBST specimens were randomly divided into two groups ( *n* = 20) according to the dentin sealing time: IDS and DDS. Each group was further subdivided into two subgroups ( *n* = 10) according to the adhesive system used for dentin sealing; iBOND self-etch adhesive (A1) and GLUMA Bond Universal (A2).

#### Immediate Dentin Sealing

Dentin surfaces of this group specimens were sealed immediately after preparation. Specimens of IDS A1 subgroup were immediately sealed using iBOND self-etch adhesive according to the manufacturer's instructions. The adhesive was agitated for 20 seconds with a brush and the dentin surface was carefully air-dried for 5 seconds with a flow of oil-free air to evaporate the solvent and water from the bonding layer. Then, adhesive was light cured for 10 seconds at a light irradiance of 1200 mW/cm ^2^ using an LED curing device (Blue phase C5 LED, Ivoclar Vivadent AG, Shaan, Liechtenstein). The LED guide tip diameter was 14 mm and was kept at zero distance with the specimen surface on curing. Prevention of an oxygen inhibited layer formation was achieved through coating of the adhesive layer with a layer of glycerin gel and light cured for 10 seconds. Meanwhile specimens of IDS A2 subgroup were immediately sealed using GLUMA Bond Universal. The adhesive was applied according to the manufacturer's instructions to the dentin surface with an applicator brush and rubbed for 20 seconds. The entire dentin surface was dried sufficiently by blowing mild air for more than 5 seconds, then the adhesive layer was isolated with glycerin gel and further light cured for 10 seconds. The specimens were temporarily restored with provisional resin discs that were fabricated through light curing of temporary restorative material (DiaTemp, DiaDent, Buk-do, Korea) for 40 seconds in a silicone mold. A chemically set temporality cement (Cavex, RW Haarlem, Holland) was used for cementing the provisional discs.

#### Simulated Pulp Pressure Mechanism

All temporally restored specimens were subjected to simulated pulpal pressure after the luting phase. 7 Ten centimeter-long semitransparent silicone tube was inserted and sealed with modeling wax through the hole formed in each tooth's pulp chamber. A dental injector was used to inject distilled water into the tubes. T-shaped pneumatic pipes were used to connect twenty tubes that were linked to the specimens with each other (Yonggao Co., Zhejiang, China). For pressure monitoring, handmade “U” manometers were mounted at the beginning and the end of the device. Adjustment of the level of air escape was performed through installing a ⅛ NPT flow regulating valve (Pneumadyne Inc., North Plymouth, Minnesota, United States) at the end of the system. An aquarium pump (OF, Z-2000, Osaka, Japan) with two outlets was attached to generate 15 cm water pressure. The specimens were restored at 0 cm H2O water pressure, then the pulpal pressure was applied 1 hour after the procedure to simulate the clinical situation in which local anesthesia produces vasoconstriction with subsequent reduction in the pulpal pressure. 15 After 7 days of specimens' storage under simulated pulp pressure, the provisional discs were removed using an excavator and dentin surfaces were cleaned by airborne-particle abrasion (CoJet, 3M ESPE) to be ready for final cementation.

#### Delayed Dentin Sealing (Control group)

After preparation of DDS group specimens, they were temporarily restored using provisional resin discs and then stored for 7 days under simulated pulp pressure as described in IDS group. Following that delay, the provisional discs were removed and dentin surfaces cleaned. Dentin surfaces were then sealed according to their assigned subgroups following the manufacture instructions as in IDS group (DDS A1; iBOND self-etch adhesive & DDS A2; GLUMA Bond Universal).

#### Fabrication and Cementation of Resin Composite Blocks

Permanent restoration of all specimens was performed through milling of cylindrical nanohybrid resin composite blocks (Grandio; shade A3, VOCO Germany) with a diameter of 12 mm and height of 4 mm using a milling machine (IMES- ICORE -250i, GmbH, Germany). The fitting surface of each block had been abraded under water cooling with 600-grit SiC paper followed by airborne-particle abrasion with 50 um aluminum oxide particles for 10 seconds to create a flat surface with standardized roughness for proper cementation. After rinsing with running water, the fitting surface of each block was air-dried and primed using a silane coupling agent for 60 seconds, then air dried before final cementation. The blocks were seated after gentle dispensing of self-adhesive resin cement on the prepared specimens. Using an especially fabricated cementation unit, a static load (1 kg for 5 minutes) was applied during block cementation.

#### Water Storage and Microtensile Bond Strength Testing

Each specimen was mounted in acrylic resin block using cylindrical Teflon mold of 15 mm diameter and 40 mm height. Half of each subgroup specimens ( *n* = 5) was measured after 24 hours and the other half was measured after 6 months of water storage in distilled water. Then, each specimen was serially sectioned perpendicular to the adhesive tooth interface into beams with a cross-sectional bonded area of ~1 mm ^2^ using a diamond saw ((IsoMet 4000; Buehler LTD). At a cross-head speed of 0.5 mm/min, tensile load of 500 N was applied till beam failure. Ten beams were selected for measuring μTBS of each specimen and recorded in mega pascal (MPa) by a computer software connected to the testing machine (Bluehill 3 Software, version 3.3 Instron, model 3345 England). Maximum tensile load was divided by the specimen cross-sectional area to obtain results in units of stress (MPa). Then, mean values were calculated for each tooth at each testing time.

### Dentin Permeability Measurement

Ten dentin discs were fabricated using IsoMet (4000 Buehler saw, United States) from the prepared specimens (one disc each). Each disc was of 1 mm thickness that was carefully examined under a stereo microscope after sectioning to ensure being free of coronal enamel or pulpal tissue. 16 Each disc's occlusal surface was marked with a permanent marker to ensure proper materials application and that the specimens were properly mounted in the filtration apparatus. All prepared specimens were held in deionized water until the procedure was completed. 17 A fluid filtration system with split-chamber hydraulic conductivity system as identified by Pashley and Galloway was utilized. 18 The fluid transport apparatus included a Teflon split chamber system, which is made up of two parts: a female and a male component that are screwed together. Two identical rubber “O” rings of 6 mm diameter were used to customize the chamber to the specimens and standardize the exposed dentin area to ensure reproducibility of the measurements. Simulation of the physiological pulpal pressure was performed as described before. 19 20

All discs were etched with phosphoric acid etching gel (Meta, South Korea) for 15 seconds to remove the smear layer prior to thorough rinsing with deionized water and slight air-drying. Air bubble displacement inside a graduated glass tube was determined in the beginning of the procedure and after 10 minutes of switching the pressure pump. Any specimen that showed any signs of dentinal blockage or leakage was excluded immediately from the experiment and replaced to end up with 10 specimens as planned. Fluid filtration (Q) was measured from the linear air bubble displacement to the nearest 0.01 mm according to the following equation 20 :

Q = displacement x cross sectional area of the pipette:

Permeability was expressed in terms of fluid filtration (Jv) where: Jv = Q/AT

Jv = fluid filtration rate in ml cm ^−2^ min ^−1^ ,

Q = fluid flow in mL,

A = dentinal surface area in cm ^2^

T = time in minutes.

Baseline fluid filtration represents the maximum fluid flow of each specimen and was randomly assigned to a value of 100% permeability. Then, the fluid flow rate was measured after smear layer creation using 600-grit sand paper discs under water irrigation and after tested adhesive application. Dentin sealing percentage of each specimen was obtained using the following equation, with each specimen serving as its own control: Permeability reduction percentage = Difference between fluid filtration rate at the baseline and after treatment protocol **/** Baseline fluid filtration rate **x** 100. 21

## Statistical Analysis

μTBS and permeability reduction percentage showed a parametric distribution when checked using Shapiro–Wilk test. Bond strength data were analyzed using Weibull analysis. Weibull parameters were calculated using maximum likelihood estimation, and 95% confidence intervals were calculated with Monte Carlo simulations. The different groups were compared at the characteristic strength (63.2% probability of failure). Independent *t* -test was used to compare between the tested groups.

### Microtensile Bond Strength Results

**Fig. 1:**
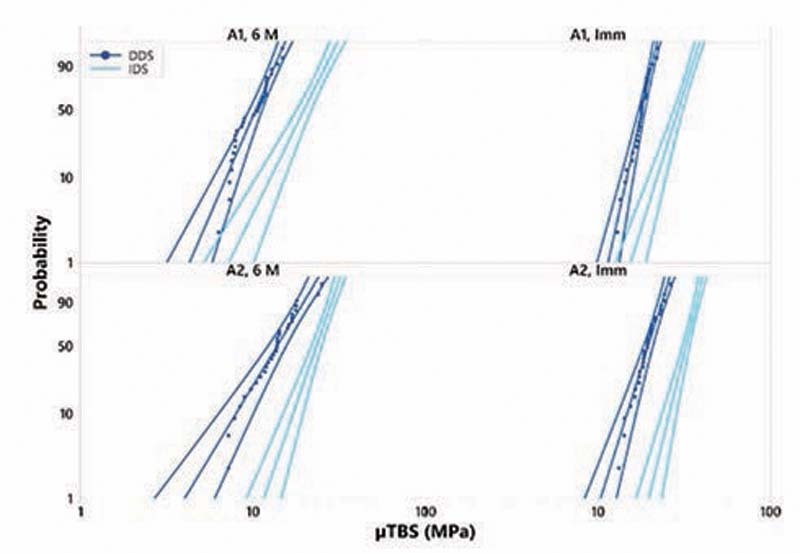
Weibull survival graph of microtensile bond strength of the tested subgroups. DDS, delayed dentin sealing; IDS, immediate dentin sealing; μTBS, microtensile bond strength.

IDS technique significantly increased the μTBS compared with the DDS for both tested adhesives after 24 hours and 6 months of water storage. GLUMA Bond Universal adhesive showed significantly higher μTBS values compared with iBOND self-etch adhesive after 24 hours and 6 months of water storage at both IDS and DDS techniques. Both materials suffered a significant reduction in the Weibull characteristic strength after 6 months of water storage in comparison to the 24 hours values ( Table 2 and Fig. 1 ).

**Table 2:** Weibull analysis results of microtensile bond strength

Water storage	Subgroup	Mean±SD	α [95% CI]	B [95% CI2]	P10 [95% CI4]
24 h	DDSA1	17.7 ± 2.2	18.6 [17.9–19.4] ^c^	9.4 [7–12]	14.7 [13.2–15.8]
24 h	DDSA2	23.1 ± 4.2	24.8 (23.2–26.5) ^b^	6 (4.5–7.7)	17.1 (14.5–19.2)
24 h	IDSA1	24.7 ± 3.6	26.2 (25–27.5) ^b^	8 (5.9–10.3)	19.8 (17.5–21.6)
24 h	IDSA2	31.9 ± 4.4	33.7 [32.2–35.3] ^a^	8.5 [6.3–11.1]	25.9 [23–28.2]
6 mo	DDSA1	10.2 ± 2.4	11.2 [10.3–12.1] ^d^	4.8 [3.5–6.2]	7 [5.7–8.1]
6 mo	DDSA2	16.6 ± 2.6	17.7 (16.6–18.7) ^c^	6.7 (5–8.5)	12.6 (10.9–14)
6 mo	IDSA1	15.9 ± 2.5	17.1 [16.2–18] ^c^	6.6 [5–8.2]	12.4 [10.8–14.1]
6 mo	IDSA2	21.9 ± 3.7	23.5 (22–25) ^b^	6.1 (4.6–7.8)	16.3 (14–18.2)

Abbreviations: DDSA1, delayed dentin sealing using iBOND self-etch adhesive; DDSA2, delayed dentin sealing using GLUMA Bond Universal; IDSA1, immediate dentine sealing using iBOND self-etch adhesive; IDSA2, immediate dentin sealing using GLUMA Bond Universal; SD, standard deviation.

Note: Different superscript letters within (α) and (P10) columns are statistically significant based on 95% confidence interval (CI). α: characteristic strength or scale of Weibull parameter. β: the shape, slope, and modulus of Weibull parameter. P10: Estimation and 95% CI at 10% probability of failure.

### Permeability Reduction Percentage Results

At a significant level of *p* = 0.05 (α=0.05), a significant decrease in the permeability reduction percentage resulted after smear layer creation in comparison to the base line values. Both tested adhesives showed significant reduction in dentin permeability percentage in comparison to the smear layer mean values. Meanwhile, insignificant difference was recorded in the permeability reduction percentage between both tested adhesives ( Table 3 ).

**Table 3:** Mean and standard deviation (SD) of permeability reduction percentage

Adhesive type	Mean ± SD of permeability reduction percentage of smear layer	Mean ± SD of permeability reduction percentage of adhesive	*p* -Value
iBond self-etch adhesive (A1)	64.78 ^a^ ±5.9	89.2 ^b^ ±3.9	0.437
GLUMA Bond Universal (A2)	66.9 ^a^ ±9.4	89.6 ^b^ ±3.2	

Note: Different letters within each row indicate significant difference, while same letters within same row indicate insignificant difference.

## Discussion

The growing popularity of CAD/CAM systems has resulted in frequent use of indirect restorations in the daily clinic. As a consequence, understanding of cementation procedure and postoperative sensitivity is a critical step that the clinician must handle before making a final decision. The traditional way of cementing indirect resin composite restorations with has been achieved by DDS after tooth preparation and temporization. Nevertheless, since the dentin is contaminated before it is bonded, and the dentin-resin hybrid layer collapses easily before it is light-cured, this technique does not provide optimum bonding conditions, resulting in reduced bond strength between the restoration and the dentin interface. 22

Hence, IDS has been used in restorative dentistry as an alternative to the traditional DDS technique in the quest for the best possible cementation protocol. The current study revealed that the IDS technique has significantly higher μTBS than the DDS technique after 24 hours and 6 months of water storage. As a result, the first null hypothesis that there is no difference in bond strength of IDS and DDS techniques either after 24 hours or 6 months of water storage was rejected. There are at least three rational explanations in the literature that support the efficacy of IDS on dentin bond strength. Owing to dentin contamination related to utilization of numerous different provisional cements to meet the practical and esthetic needs of the patient, DDS technique can result in a significant reduction in bond strength. 23 Thereby, the first explanation is related to bonding to freshly cut dentin in IDS technique that is the ideal substrate for dentin bonding. 10 Second, the prepolymerization of dentin adhesive could explain the improved bond strength values of IDS technique. The failure of the unpolymerized dentin–resin hybrid layer attributed to pressure exertion during restoration seating may explain these findings. 24 25 Third, IDS provides stress-free dentin bond development, as the literature has shown a substantial increase in bond strength over a 1 week span. 26

Regarding the adhesive effect on the bond strength, the current study also revealed that GLUMA Bond Universal recorded significantly higher bond strength values than iBOND self-etch adhesives after 24 hours and 6 months of water storage. Consequently, the second null hypothesis that there is no difference in bond strength exists when self-etch mode of universal adhesive and a self-etch adhesive used for IDS and DDS when tested after 24 hours and 6 months of water storage was rejected. This may be attributable to the fact that GLUMA Bond Universal contains a 10-MDP acidic monomer in addition to the 4-methacryloyloxyethyltrimellitate anhydride (4 META) in their ingredients, both of which have been shown to interact chemically with hydroxyapatite. 27 On the other hand, the iBOND self-etch adhesive contains 4META monomer only. Since 10-MDP generates a strong nanolayer along the adhesive interface based on the chemical bond with dentin's hydroxyapatite, the high bond strength related to GLUMA Bond Universal can be attributed to stable MDP-Ca salt deposition. 28 29

Unfortunately, after 6 months of water aging, both tested adhesives showed reduction in bond strength values. It is conceivable that their chemical interaction, which has been shown to deteriorate by aging, could be the reason for their reduced values. 30 Another issue with decreased bond strength after water aging is the possibility of phase separation due to the vapor pressure differences between the acetone and water in both tested adhesives. 31 32 Furthermore, as the adhesive's acidity increases, problems with water permeability worsen, leaving water-filled nanospaces at the interfacial layer. Water leads to both collagen fibril degradation and composite plasticization, resulting in accelerated hybrid layer deterioration and, as a result, a reduction in dentin bond strength over time. 33

Regarding dentin permeability evaluation, assessment of hydraulic conductance has been documented to be an appropriate method for evaluating dentinal tubule occlusion. 34 35 It also facilitates the comparison between various treatment protocols by providing objective and quantitative results. 36 Since the adhesion mechanism of self-etch adhesives includes incorporating the smear layer into the adhesive interface 37 in the current experiment, a new smear layer was developed on the surface of each dentin disc before incorporating the adhesives. By sealing the dentin surface with an acid-resistant hybrid coating and occluding the tubule orifices with resin tags, self-etch adhesives were shown to be efficient in decreasing dentin permeability, 38 which was already demonstrated in our research. Even so, none of the tested adhesives in the current research displayed absolute dentin sealing (100%); both revealed effective dentin sealing without any significant difference between them. Based on these findings, the third null hypothesis that there is no difference in the ability of the tested adhesive systems to reduce the dentin permeability was accepted. Consequently, clinicians can benefit from reduced sensitivity during temporization and after final cementation of indirect resin composite restorations without having to worry about bond strength being compromised.

## Conclusion

Under the limitations of the present study, it can be concluded that the IDS technique using self-etch mode of the universal adhesive is an effective strategy for improving the final bond strength of CAD/CAM resin composite restorations and reducing post-cementation sensitivity.
